# Advancing cancer immunotherapy: from innovative preclinical models to clinical insights

**DOI:** 10.1038/s41598-024-51704-5

**Published:** 2024-01-12

**Authors:** Andrew W. Craig, Hermann B. Frieboes, Paula A. Videira

**Affiliations:** 1 Department of Biomedical and Molecular Sciences, Queen’s University, Kingston, ON Canada; 2 Cancer Biology and Genetics Division, Queen’s Cancer Research Institute, Kingston, ON Canada; 3 Department of Bioengineering, University of Louisville, Louisville, KY USA; 4 U of L Health – Brown Cancer Center, University of Louisville, Louisville, KY USA; 5 Center for Predictive Medicine, University of Louisville, Louisville, KY USA; 6 UCIBIO – Applied Molecular Biosciences Unit, Department of Life Sciences, NOVA School of Science and Technology, Universidade NOVA de Lisboa, Caparica, Portugal; 7 Associate Laboratory i4HB - Institute for Health and Bioeconomy, NOVA School of Science and Technology, Universidade NOVA de Lisboa, Caparica, Portugal

**Keywords:** Cancer immunotherapy, Cancer models

## Abstract

The rapid expansion of cancer immunology and immunotherapy builds upon the success of early immune checkpoint inhibitors (ICI) and chimeric antigen receptor T cells for some cancer types. Many gaps still exist, however, in the scientific knowledge of immune dysfunction in the tumour microenvironment and predicting clinical immunotherapy response to allow more cancer patients to benefit from immunotherapy. The Cancer Immunotherapy Collection within *Scientific Reports* describes pioneering preclinical and clinical studies addressing these concepts, representing significant insights and breakthroughs in the field.

The studies featured in this Collection showcase the innovative endeavours in cancer immunotherapy using preclinical models and clinical data. The diverse works explored novel avenues and challenges in therapies such as cytokines, cellular therapies and small molecules. In metastatic castration-resistant prostate cancer, Korentzelos and collaborators investigated the ability of interferon-γ (IFNγ) to increase major histocompatibility class-I (MHC-I) and PD-L1 expression, facilitating antigen presentation and immune checkpoint blockade therapy. Combining IFNγ with paclitaxel mitigated metastatic disease in a murine tumour model, suggesting promise for combinatory regimens^1^. The functional limitations of natural killer (NK) cells in glioblastoma patients were unravelled by Sönmez and collaborators, who showed that the blockade of the inhibitory KIR receptors and IL-2 stimulation bolsters NK responses^2^. This study sheds light on new approaches for cancer immunotherapy to glioblastoma. Diverging from this approach, yet converging in the quest for innovative therapies, Panyam and collaborators demonstrated that novel small molecule TLR7/8 agonists induce robust pro-inflammatory cytokine and enhance NK cell-mediated antibody-dependent cellular cytotoxicity in their models^3^. These agonists show promise in augmenting the anti-cancer efficacy of monoclonal antibodies, offering new dimensions to combinatory immunotherapy.

A novel approach to targeted immunotherapy for triple-negative breast cancer (TNBC) was introduced by Lin and collaborators, who identified tropomyosin receptor kinase B (TrkB) as a selective TNBC cell marker for refined antibody–drug conjugates to enhance treatment precision^4^. This study both opens avenue to explore applications of refined antibody–drug conjugates and addresses the challenge associated with the lack of targets for TNBC. In a parallel exploration, the expression of PD-1 on murine melanoma cells was validated by Martins and collaborators who, in doing so, provide a foundation for further investigations into PD-1 biology and implications in immune checkpoint therapy^5^. Takahashi and collaborators also pursued a preclinical angle, introducing a novel humanized mouse model to evaluate ICI targeting PD-1 by merging an immunocompromised mouse strain with two IgG receptor knockouts^6^. The authors showed improved tumour growth restriction by Nivolumab, prompting future studies testing other immunotherapies in this model.

The pathways leading to the recruitment of Tissue-resident memory (Trm) CD8 T cells in mouse pancreatic cancer models were evaluated by Gough and collaborators, who found that the activation of CD8 Trm is dependent on CD40L signalling in tumour-draining lymph nodes^7^. These findings inform new strategies for harnessing Trm cells' potential in cancer treatment. In another study, Trinklein and collaborators reported novel bispecific antibodies that act as an agonist of IL-2 signalling in cytotoxic T cells and NK cells without activating regulatory T cells. One of these bispecific antibodies activates STAT5 signalling and expanded CD8 T cells in a monkey model, with no overt toxicities observed^8^. This new approach to harness the desired effects of the IL-2 pathway is primed for further testing in various cancers.

In exploration at the intersection of veterinary and human oncology, Dias and collaborators showed the potential of canine lymphoma (cNHL) as a preclinical model for testing anti-CD20 immunotherapies in B-cell malignancies. A novel single-domain antibody that binds to both human and canine CD20 and can be used in therapeutic and diagnostic approaches thus advancing both veterinary and human oncology^9^.

Amengual-Rigo and Guallar presented NetCleave tool, an open-source and retrainable algorithm predicting C-terminal antigen processing for both MHC-I and MHC-II pathways^10^. This enhances our ability to understand and potentially manipulate antigen processing, thereby influencing immune responses^11^. A different tool was presented by Mehdizadeh and colleagues for simulating myeloid-derived suppressor cells (MDSC) depletion in a mouse model of aggressive tumours. The computational simulations suggest that vaccination with a small number of tumour cells in combination with MDSC depletion elicits an effective anti-tumour immune response and tumour dormancy^12^.

The studies using clinical data featured in this Collection exemplify efforts to eventually translate research on biomarkers and mechanisms of immunotherapy responders or non-responders in clinical trials or clinical care, thus offering insights into improving cancer treatment strategies. Kauffmann-Guerrero and collaborators investigated the potential of inflammation and cytokine profiles as biomarkers for non-small-cell lung cancer patients receiving ICI and revealed inflammation markers that dictate response to ICI treatment^13^. This study highlights the limitations of relying solely on PD-L1 expression and emphasizes the importance of inflammatory biomarkers for predicting treatment response. The analysis of immune changes in multiple myeloma patients receiving ICI and the immunomodulator pomalidomide in Phase 1b trial (NCT02616640) reported by Newhall and collaborators emphasized transcriptome changes consistent with favourable immunomodulation^14^, but also the risk of increasing autoimmune response and adverse events, as evidenced by other ongoing trials. In a parallel exploration, Mendoza and co-workers detailed patients’ symptom burden in early-phase trials for rare solid tumours treated with immunotherapy. This prospective longitudinal study showed the distribution of high immunotherapy-specific symptom burden^15^, and the results may help inform the planning of future symptom interventional clinical trials for patients receiving ICI.

Continuing the quest for the efficacy of combination therapies, the real-world outcomes for metastatic non-small cell lung cancer patients treated with first-line Pembrolizumab plus chemotherapy were compared by Velcheti and collaborators to the clinical trial that led to the approval of this combined immunotherapy. This study provided substantial evidence of outcomes from combination therapy in a more heterogeneous patient cohort and clinical care setting^16^. In a departure from traditional biomarkers, Naing and collaborators brought us the associations between microbiome composition and fatigue in advanced cancer patients. The authors revealed microbiome-associated bacteria negatively and positively associated with fatigue severity^17^, uncovering potential insights into patient well-being and treatment outcomes.

In searching for novel immune molecular classification, Yu and colleagues applied a non-negative matrix factorization algorithm and subdivided colorectal cancer into immune classes based on the immunocyte infiltration and enrichment of immune response-associated signatures. The immune-suppressed subclass had the worst overall prognosis, while patients within the immune-activated subclass showed better prognosis and response to anti-PD-1 therapy^18^. Finally, from a different perspective, Krishnan and colleagues proposed the GaWRDenMap framework utilizing geographically weighted regression and a density function-based classification model that discriminates between chronic pancreatitis, pancreatic ductal adenocarcinoma (PDAC) and intraductal papillary mucinous neoplasm at both the subject- and image-levels^19^. It could also reasonably discriminate between PDAC. These results indicate a potential difference in the spatial arrangement of epithelial and immune cells in the pancreas can have diagnostic significance.

## Conclusions

This Advancing Cancer Immunotherapy Collection in *Scientific Reports* embodies diverse studies pushing the boundaries of cancer immunology and treatment. These papers deepen our understanding of immunotherapeutic mechanisms and present novel strategies and perspectives to advance cancer treatment outcomes. The research findings within this Collection include innovations and highlight the power of collaborations across disciplines, paving the way to expanding the application of cancer immunotherapy so more cancer patients will benefit.
